# Correlation of Body Mass Index with Pelvis and Lumbar Spine Alignment in Sagittal Plane in Hemophilia Patients

**DOI:** 10.3390/medicina55100627

**Published:** 2019-09-24

**Authors:** Klaudia Zawojska, Agnieszka Wnuk-Scardaccione, Jan Bilski, Ewa Nitecka

**Affiliations:** Department of Ergonomics and Physiology of Physical Exercise, Faculty of Health Sciences, Jagiellonian University Collegium Medicum, 31-531 Cracow, Poland; agnieszka90.wnuk@uj.edu.pl (A.W.-S.); jan.bilski@uj.edu.pl (J.B.); ewa.nitecka@uj.edu.pl (E.N.)

**Keywords:** hemophilia, body mass index, lumbar-pelvic complex, spinopelvic alignment, hemophilic arthropathy

## Abstract

*Introduction:* Concern about weight gain among people has been high due to negative health consequences in addition to the increasing prevalence of the problem. Overweight and obesity also occur in patients with hemophilia. Analysis of literature shows that increased body weight might have a biomechanical effect on the spatial orientation of the pelvis and the lumbar spine. The aim of this study was to determine the correlation between body mass index (BMI) and the parameters characterizing the alignment of the sacrum (SS, sacral slope), the pelvis (PT, pelvic tilt; PI, pelvic incidence) and the angle value of lumbar lordosis (LL, lumbar lordosis) assessed in the sagittal plane among patients with hemophilia. *Materials and methods:* A total of 49 patients were subjected to the study, 23 of whom met the inclusion criteria. Body weight and height were measured. Measurement of the angle values of indicators characterizing the position of the lumbar–pelvic complex was established based on X-ray imaging analysis. *Results:* Analysis of the correlation between the BMI and sacral, pelvic, and lumbar indicators evaluated in the sagittal plane in the study group of patients with hemophilia showed a correlation between BMI and SS (*r* = 0.48). SS values were significantly and positively related to PI (*r* = 0.6; *p* = 0.002) and LL (*r* = 0.46; *p* = 0.02). The results obtained indicate the BMI relationship with the setting of the sacrum in the sagittal plane (SS). After adjusting for the knee flexion contracture, the correlation on the border of significance (*b* = 0.73, *p* = 0.07) between the body mass index and the spatial orientation of the pelvis and the spine was revealed. *Conclusion:* We hypothesize that increased body weight among people with hemophilia might have an effect on the positioning of the lumbosacral region. Therefore, it is believed that preventing obesity among people with hemophilia can contribute to a smaller number of intra-articular hemorrhages and better orthopedic condition of the limb joints, and thus could avoid changes in the lumbosacral region as well as their consequences.

## 1. Introduction

Excess body weight and obesity are currently being considered as major health problems affecting human beings worldwide [1]. They are associated with not only metabolic and cardiovascular diseases but also with a number of disorders of the locomotor system, resulting from increased pressure on the articular surfaces and changes in the alignment of the spine, the pelvis, and the lower extremities [2,3]. The body mass index (BMI) is used to characterize weight to height ratio in adults [4]. Four categories are identified; a person is underweight if his/her BMI is in the range of 15 to 19.9 kg/m^2^, normal weight if the BMI is 20 to 24.9 kg/m^2^, overweight if the BMI is 25 to 29.9 kg/m^2^, and obese if the BMI is 30 to 35 kg/m^2^ or greater [5].

Hemophilia is a rare genetic disorder associated with chromosome X in which a deficiency of clotting factor (VIII in hemophilia type A and IX in hemophilia type B) makes the bleeding time longer [6]. The severity of bleeding depends on the level of activity of the deficient factor [7]. The clinical picture of hemophilia is dominated by complications within the musculoskeletal system [8,9,10]. The majority of patients present arthropathic changes in multiple joints, both those affected by hemorrhages as well as the neighboring ones. Biomechanics disorder and pathological overload in the musculoskeletal system cause the adaptation of the entire motion system [11].

Overweight and obesity also occur in patients with hemophilia [12]. This particular group of patients is characterized by a low degree of physical activity, which is most likely a consequence of poor joint condition. Also, a very important factor that limits activity in patients with hemophilia is the fear of bleeding [13]. However, it is a vicious circle leading to further weight gain and consequent deterioration of articular structures and disruption of biomechanics, especially of the lower extremities and the lumbosacral complex.

Many authors attempted to determine the average values of spine lordosis and parameters of pelvic alignment [14,15,16] while looking for the correlation between clinical results and imaging studies. However, everyone consents to the fact that these anthropometric indicators are very diverse in the population, and it is difficult to establish normative values unequivocally. Noshchenko et al. [17] conducted a systematic review along with a meta-analysis, based on which they presented optimal values for spinal and pelvic indicators in people without disease or spinal deformity. They are: 37° (22–53) for the lumbar lordosis (LL) index measured by the Cobb method from the upper surface L1 to the lower surface L5 or 54.6° (42–67) for LL measured by the Cobb method from the upper surface L1 to the upper surface S1; 50.6° (39–62) for pelvic incidence (PI); 37.7° (28–48) for sacral slope (SS); 12.6° (3–22) for pelvic tilt (PT). Analysis of literature shows that increased body weight may have a biomechanical effect on the spatial orientation of the pelvis and the lumbar spine [18]. However, some researchers do not agree on this issue, and thus there are publications in which there is almost no correlation between BMI and the alignment of the lumbar–pelvic complex [19]. 

Referring to the register of the Institute of Hematology and Transfusiology, Warsaw, Poland, there are approximately 2600 people suffering from hemophilia in Poland, including about 2300 people with type A hemophilia, out of which approximately 1100 people have a severe form of this condition without the presence of an inhibitor, and approximately 150 people have a severe form with the presence of an inhibitor. There are about 400 people suffering from type B hemophilia, out of which approximately 200 patients have a severe form of this condition without the presence of an inhibitor, and several patients have a severe form with the presence of an inhibitor. 

The aim of this study was to determine the correlation between BMI and the parameters characterizing the alignment of the sacrum, the pelvis, and the angle value of lumbar lordosis assessed in the sagittal plane in patients with hemophilia. We hypothesize that increased body weight among people with hemophilia affects the positioning of the lumbosacral region. The results obtained may be significant in terms of prevention of the development of overweight as well as structural and biomechanical changes in the limb joints and the lumbosacral complex in hemophilia patients. 

## 2. Materials and Methods

The article presents the results of one part of the study carried out in the group of male subjects suffering from hemophilia, who successively presented themselves to the Hematology Clinic, University Hospital, Cracow, Poland throughout 2015–2016. The follow-up study was initiated after approval of the Bioethics Commission of the UJCM (Jagiellonian University Collegium Medicum) No. KBET/254/B/2014. A total of 49 patients were subjected to the study, 23 of whom met the inclusion criteria, of which the most important were flexion contracture in the knee joint > 5º and lack of flexion contracture in the hip joints. Candidates who did not meet the above criteria were excluded from the study, as were candidates who had contracture in the knee joint due to a cause other than hemophilic arthropathy, who underwent endoprosthetics in the knee joint (which would be considered in the study), who underwent a surgery in the lumbar spine, or who were diagnosed with an active tumor condition.

**Figure 1:**
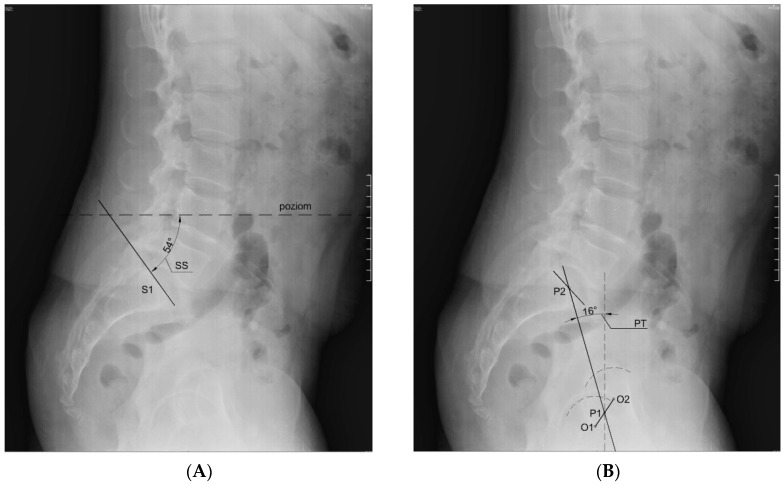

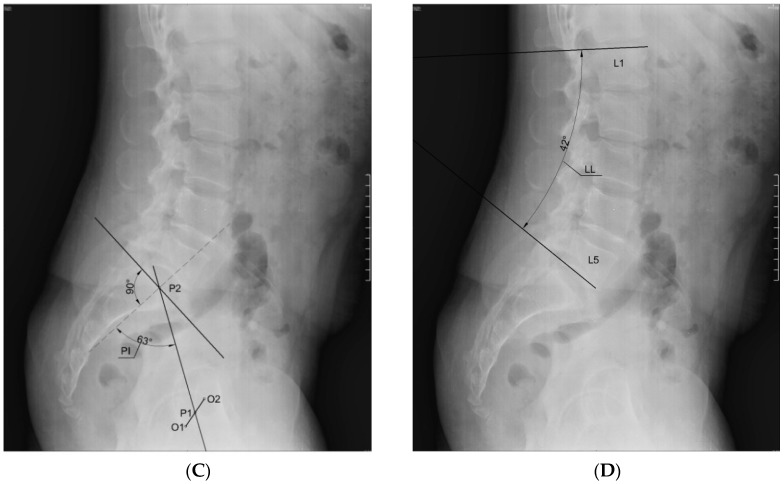
Method of plotting of the angle denoting: **A**—the sacral slope (SS) in the sagittal plane; **B**—the pelvic tilt (PT) in the sagittal plane; **C**—the pelvic incidence (PI) in the sagittal plane; **D**—the value of curvature of the lumbar spine (lumbar lordosis, LL) in the sagittal plane. Source: archive of own images.

The mobility range test of the knee joints was performed first (goniometer MoVes 08-030202). The results of the test determined whether or not the candidate was included in the study. Secondly, body weight and height were measured (portable height meter Leicester Tanita HR 001, Tanita; weight measuring device Tanita BC-543, Tanita), and the BMI was calculated. In the next step, the researchers performed an X-ray in the lateral projection in standing position with the visualization of the lumbar spine, the pelvis, and the femoral heads. The subjects were asked to take a relaxed stance with their hands crossed on their chest (fists on the clavicles). If the flexion contracture occurred on the right side, the subjects were asked to position themselves with their right side to the radiation source, and if the flexion contracture occurred on the left side, they were asked to position themselves with their left side to the source of the radiation. The subjects kept their footwear for the X-ray test. If the subjects wore shoe inlays on a daily basis, the X-ray was done with them. The distance from the radiation source was 100 cm. The radiation beam was targeted at the L3 vertebra. Measurement of the angle values of indicators characterizing the position of the lumbar–pelvic complex—SS, PT, PI, and LL (Figure 1)—was established on the basis of X-ray imaging analysis. Measurements were made using AutoCAD LT 2013 software (Autodesk). The correctness of plotted angles was assessed by radiologist (MS) using the mediCAD program (mediCAD Hectec GmbH).

### Statistical Analysis

The distribution of qualitative variables was described by indicating absolute and relative values. The distribution of quantitative traits was described by indicating mean values and standard deviation or by median and lower (Q1) and upper (Q3) quartile in cases where distribution of traits deviated from normal distribution. The compatibility of the variable with the normal distribution was evaluated using the Shapiro–Wilk test. The distributions of BMI and the parameters characterizing the alignment of the sacrum, the pelvis, and the angle value of lumbar lordosis were normal. Due to this fact, the Pearson correlations were used to analyze the correlation between BMI and the indicators characterizing pelvic and lumbar alignment in the sagittal plane as well as to analyze the correlation between individual lumbosacral indicators. All analyses were performed using *Statistica v. 12* software (StatSoft Polska, Cracow, Poland), and α = 0.05 was adopted as the significance level for bilateral tests.

## 3. Results

The study included 23 male subjects who met the inclusion criteria. Out of 23 subjects, 21 (91.3%) suffered from type A hemophilia, and there were two (8.7%) with type B of this condition. Twenty subjects (87%) suffered from a severe form of hemophilia, and three subjects (13%) had a moderate form of this condition. The average age of the participants was 39.6 (SD = 8.58 years; range: 20–52 years). The average BMI was 24.6 kg/m^2^ (SD = 4.22 kg/m^2^; range: 18.1–32.5 kg/m^2^). In total, 13% of patients were obese, 35% were overweight, 44% had normal body weight, and 9% were underweight. The median number of intra-artricular hemorrhages per year was 10; Q1 = 3, Q3 = 20, range: 0–60. Nineteen patients were on prophylactic treatment, three were on “on demand” treatment, one of the patients, due to liver transplantation, stopped taking the coagulation factor and had no intra-artricular hemorrhages.

Table 1 provides descriptive statistics for the values of indicators characterizing the alignment of the sacrum, the pelvis, and the lumbar spine.

Analysis of the correlation between BMI and sacrum, pelvis, and lumbar spine alignment indicators evaluated in the sagittal plane in the study group of patients with hemophilia (Table 2) showed a correlation between BMI and SS (*r* = 0.48, *p* = 0.02). 

**Figure 2:**
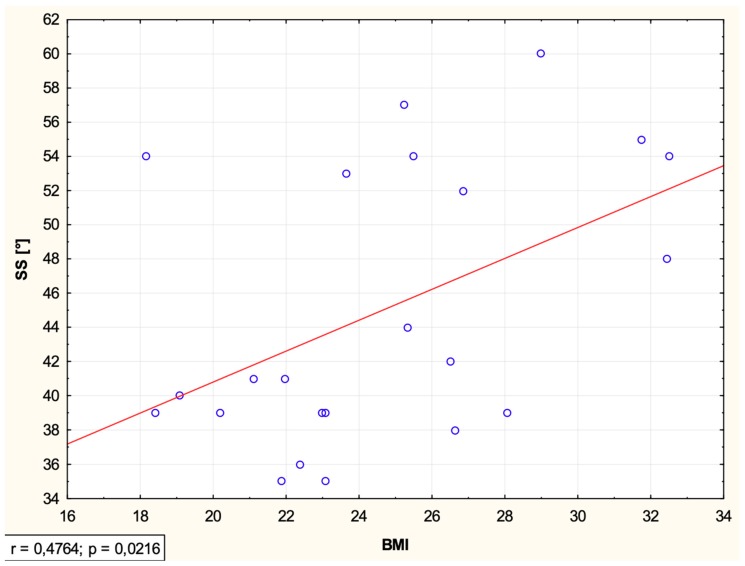
Scatter plot showing the relationship between BMI and the sacral slope (SS) in hemophilia patients.

The scatter plot (Figure 2) shows the relationship between BMI and the sacral slope (SS). After adjustment for the flexion contracture, the relationship between BMI and SS was on the border of significance (*b* = 0.73, *p* = 0.07).

## 4. Discussion

In this study, we evaluated the correlation between BMI and lumbo-pelvic indicators in the sagittal plane in a group of adult patients with hemophilia and a flexion contracture of the knee joint. A moderate correlation between BMI and SS (*r* = 0.48, *p* = 0.02) was shown, which means that higher values of BMI were associated with greater sacral slope. 

Obesity is associated with increased morbidity and mortality from ischemic heart disease, stroke, diabetes, chronic renal failure, respiratory disease, and several types of cancer [20]. It also significantly contributes to the development of osteoarthritis, which, in combination with joints hemorrhages, may aggravate clinical symptoms of hemophilia. As demonstrated by Wong and associates [21], in the group of boys with hemophilia aged 2–5, the risk of obesity was higher compared to boys of the same age in the general population. The prevalence of obesity in other age groups as well as in adult hemophilia patients was similar to the healthy population. A nationwide study conducted in the Netherlands in 1992 and 2001 showed that, over almost 10 years, the percentage of overweight hemophiliacs increased from 27% to 35%, while the percentage of obese hemophiliacs doubled (from 4% to 8%) [22]. These results are comparable to the general population, while alarming was the fact that the number of obese boys with hemophilia tripled at that time. In the present study, 13% of patients were obese, 35% were overweight, 44% had normal body weight, and 9% were underweight. These results are therefore very similar to those presented in the Dutch study. Since the numbers are disturbing, it seems necessary to educate patients in terms of healthy nutrition and proper physical activity.

Researchers agree that obesity is an important factor contributing to the development of gonarthrosis [23,24]. Mezhov et al. [25] carried out a systematic review of the literature on the impact of obesity on the cartilage of the knee joint. This analysis showed a consistent, destructive effect of high BMI leading to degeneration and damage of the articular cartilage. The study of Bierre-Rafi and associates [26] was aimed to find correlation between obesity and limitations in everyday life, occurrence of hemorrhages, and the use of anti-hemophilic factor (AHF). The study was carried out in a group of 15 patients with obesity compared to 15 patients with normal body weight. Compared to the control group, the patients with obesity obtained a significantly lower results in the Hemophilia Activity List (HAL) questionnaire, which was mainly due to impaired lower limb function. In obese patients, joint hemorrhages were significantly more common, and a higher supply of coagulation factor concentrate was required. Similar conclusions were drawn from a case study of a patient with obesity who, following a four month body weight reduction program, achieved a significant improvement in the number of joints hemorrhages, which indicates the correlation between the BMI index and the risk of bleeding as well as the resulting arthropathy [27]. Although it is difficult to draw conclusions based upon a description of one case, it seems that body weight control in patients with hemophilia is very important, since the increased pressure on articular surfaces due to overweight or obesity combined with hemorrhages may accelerate the development of hemophilic arthropathy. 

The study of Romero-Vargas et al. [19] assessed how the BMI index may be related to central obesity and spino-pelvic indicators. Spino-pelvic indicators (PI, PT, SS, LL) were evaluated using radiographs of the lumbar spine and the pelvis performed in the lateral projection. Increased values of these indicators were observed in both BMI and central obesity groups, but these differences did not reach the level of statistical significance. The range of BMI in our sample was: 18.1–32.5 kg/m^2^ (which covers normal, overweight, and obese). We obtained positive relationships between BMI and SS (*r* = 0.48, *p* = 0.02); higher values of BMI were associated with higher values of SS. However, after adjusting for the knee flexion contracture, the relationship between BMI and SS was on the border of significance (*b* = 0.73, *p* = 0.07). Contrary to the reports of Romero-Vargas et al., in our study, the values of the indicators PT and LL decreased as BMI values increased (none of these values reached the statistical significance level). We assume, however, that the values of these indicators in the group of healthy people cannot be compared to the group of people with hemophilia. To our knowledge, there are no studies in the group of patients with hemophilia to which we could relate the obtained results.

Intra-articular bleeding causes destruction of the joint structures, leading to disorders at the level of structure (pain in the joint, limitation of mobility) as well as function (functional shortening of the limb resulting from flexion contracture, deformation or valgus/varus positioning, limitation of joint flexion, proprioception disorder). Flexion contracture in the knee joint, which was the main inclusion criteria, first affects the adjacent joints, i.e., ankle and hip joints, and both of these joints position themselves in the flexion position. We performed an analysis with BMI and the knee flexion contracture as independent variables. After adjustment for the flexion contracture in the knee joint, the relationship between BMI and SS was on the border of significance (*b* = 0.73, *p* = 0.07) in hemophilia patients; however, the direction of the relationship and the strength of the relationship were similar. Subsequently, the changes involve the pelvis. In the frontal plane, the pelvis is tilted towards the limb with the contracture. In the sagittal plane, when standing, the pelvis is in the backward tilt, and during walking, it is tilted forward. In the transverse plane, the pelvis rotates forward on the side of the contracted limb. Changes in the pelvic position entail changes in the curvature of the spine, causing pathological overloads in its structures. In this study, no relationship was found between the BMI and indicators characterizing the position of the pelvis or the lumbar spine in the sagittal plane. The next link in this chain is the upper limbs—the arm on the contractured side bends towards it (frontal plane) and protrudes on the same side. The changes described above also affect the work of the muscular system. Lack of or small range of motion in the joint cause atrophy of the muscles responsible for it, while others are overloaded. The resulting compensation schemes also result in an improper load distribution in the muscular system, consolidating them and leading to pain and disability [28,29].

This study was limited by a low number of subjects, which could affect the obtained results. In order to confirm the obtained correlation, a larger group of subjects should be evaluated. The presented results should be related to a healthy control group. Due to the lack of access to the device allowing comprehensive visualization of the spine, the pelvis, and the lower limb, measurements of the lower limb were made using a goniometer followed by a lateral X-ray of the spine and the pelvis. The fact that the measurements and the roentgenogram were not performed simultaneously could affect the investigated correlations.

## 5. Conclusions

In patients with hemophilia who also have a flexion contracture in the knee joint (joints), the BMI indicator seems to represent a predisposing factor for higher values of the sacral slope (SS); however, the relationship between BMI and SS was on the border of significance. Obtained results draw attention to the problem of increased body weight and its impact on spatial orientation of the pelvis and the spine in this group of patients as well as deterioration of articular structures and disruption of biomechanics, especially of the lower extremities and the lumbo-pelvic complex. Therefore, it seems necessary to educate hemophilia patients in the area of proper nutrition habits and the importance of physical activity. 

## Figures and Tables

**Table 1:** Distribution of indicators of the alignment of sacrum, pelvis, and lumbar spine in the group of male participants with hemophilia (*n* = 23).

Variable	Mean	SD
**SS [°]**	44.95	8.00
**PT [°]**	10.04	8.25
**PI [°]**	53.86	10.84
**LL [°]**	47.30	12.33

N—number of subjects, SD—standard deviation, Me—median, Q1—lower quartile, Q3—upper quartile.

**Table 2:** Pearson’s correlation between variables characterizing pelvic and lumbar spine alignment in the group of male patients with hemophilia (*n* = 23).

		BMI	SS	PT	PI	LL
**BMI [kg/m^2^]**	**R**	1	**0.476**	−0.074	0.305	−0.004
	**P**		**0.022**	0.736	0.157	0.986
**SS [°]**	**R**		1	0.064	**0.602**	**0.467**
	**P**			0.772	**0.002**	**0.025**
**PT [°]**	**R**			1	**0.757**	0.227
	**P**				**0.000**	0.297
**PI [°]**	**R**				1	**0.502**
	**P**					**0.015**

BMI—body mass index, N—number of subjects, *r*—correlation coefficient, *p*—limit level of significance, statistically significant values (*p* < 0.05) are in bold.
